# Rationale, design and methodology for a **P**rospective **R**andomized Study of graft patency in **O**ff-pump and On-pump **M**ult**I**-Vessel coronary artery bypas**S S**urgery (PROMISS) using multidetector computed tomography

**DOI:** 10.1186/1745-6215-9-44

**Published:** 2008-07-17

**Authors:** Miguel Sousa Uva, Fernando Matias, Sara Cavaco, Manuel Pedro Magalhães

**Affiliations:** 1 Departamento da Circulação, Serviço de Cirurgia Cardíaca, Hospital da Cruz Vermelha, Lisboa, 1549-008, Portugal; 2 Division of Behavioural Neurology and Cognitive Neuroscience, University of Iowa, Iowa City, USA

## Abstract

**Background:**

Off-pump coronary artery bypass grafting has been accused of possibly compromising graft patency. Sixteen slice computed tomography has shown good diagnostic accuracy in the assessment of coronary bypass graft patency when compared with conventional coronary artery angiography and is less invasive. The study hypothesis is that coronary artery bypass grafting (CABG) performed without cardiopulmonary bypass (Off-Pump) has equivalent early graft patency as if performed with cardiopulmonary bypass (On-Pump) and may have reduced complication rate.

**Methods/Design:**

The **P**rospective **R**andomized Comparison of **O**ff-Pump and On-Pump Mult**I**-vessel Coronary Artery Bypas**S S**urgery (PROMISS) is a controlled, single blinded, single centre clinical trial, comparing early graft patency using 16-slice computed tomography in patients with multi-vessel coronary artery disease operated either without or with extracorporeal circulation. Inclusion criteria are multivessel disease with an indication for first time, isolated, non emergent coronary artery bypass grafting with a minimum of three distal anastomoses. Secondary end points are peri-operative mortality, combined morbidity, length of stay, neuro-cognitive testing at 6 weeks and adverse events, stress test and quality of life at 6 months and one year. The sample size of one hundred and fifty patients was calculated in order to enable the detection of a 5% difference in graft patency, with 80% power, considering a minimum of 3 distal anastomoses per patient. Enrolment started in April 2005 and ended July 2007 with study closure in July 2008.

**Conclusion:**

The PROMISS trial aims to shed new light on the effect of Off-Pump as compared to On-Pump coronary artery bypass surgery on graft patency, assessed by multidetector computed tomography, in unselected patients with multivessel coronary artery disease.

**Trial Registration:**

Current Controlled Trials ISRCTN58800729

## 1. Background

Coronary artery bypass surgery is a proven and effective mode of treatment for ischaemic heart disease. It provides relief of symptoms and increases survival, in particular in patients with left main disease and two or three vessel disease including the proximal left anterior descending artery [1].

Coronary artery bypass grafting (CABG) can be performed in several ways but in the majority of cases it is still performed with the assistance of cardiopulmonary bypass (CPB), aortic cross clamping and cardioplegia induced cardiac arrest ("on-pump" or conventional method). Coronary artery bypass grating can also be performed without CPB ("off-pump" or OPCAB) and this method's application varies from around 20% in the USA and Europe to more than 50% in India and Japan.

As a consequence of increased life expectancy and a more widespread use of coronary angiography with exponential growth of percutaneous coronary interventions in ischaemic heart disease, patients proposed for CABG are older and suffer from more co-morbid conditions. In spite of this, continuous incremental improvements in peri-operative patient management, anaesthetic and surgical techniques have led to better results [2]. However, morbidity is still significant in certain patient subsets and off-pump coronary artery bypass surgery saw its development in the nineties with the rationale of reducing some of the cardiopulmonary bypass related complications [3]. Indeed, although CPB is usually well tolerated in the majority of cases, it can be responsible for complications and death, particularly in older and sicker patients with co-morbid conditions. Cardiopulmonary bypass may be a source of distal embolization by aortic canulation and manipulation and causes myocardial ischaemia-reperfusion injury through cardioplegic arrest. CPB causes also a systemic inflammatory response and increases the need for blood transfusion by hemodilution and coagulation disturbances [4].

Most studies show that OPCAB is associated with less cardiac, pulmonary, renal or neurologic complications and reduces blood transfusion and hospital stay compared with "on-pump" coronary bypass surgery [5-7]. However, there is still some debate about the type and degree of morbidity reduction achieved by OPCAB [8]. For example, neurocognitive decline is one of the most frequent sequelae of cardiac surgery but there is still no consensus whether OPCAB is superior to conventional CABG with respect to this complication [8]. Neuropsychological evaluation is based on a battery of tests evaluating memory, learning, attention, psychomotor speed, dexterity and diverging results between studies, may be due to different assessment protocols, different timings, statistical tools used to define a decline and absence of control groups.

In general, there has been difficulty in accumulating scientific evidence for OPCAB benefit, due to low incidence of events (mortality, stroke, myocardial infarction), therefore requiring very large samples or high risk populations to demonstrate a statistically significant improvement [9].

On the other hand, "off-pump" CABG has been accused of compromising completeness of revascularisation and graft patency by its increased technical difficulty, particularly in grafting the left ventricular lateral wall [10]. Indeed, a learning curve applies in OPCAB, and its benefits can be expected only with the strict application of principles and rules and a sustained practice without which results can be inferior to those obtained with conventional "on-pump" CABG. The same applies to long term results which depend on completeness and quality of revascularisation.

Coronary artery bypass grafting has an important role in the treatment of coronary artery disease as shown by series with long term results [11]. However, increased survival and symptoms relief are directly related to graft patency. Consequently, it is important that new, less invasive methods reduce the early risk of the procedure but also ensure the same revascularisation quality as the conventional technique.

The justification for the**P**rospective **R**andomized Comparison of **O**ff-Pump and On-Pump Mult**I**-vessel Coronary Artery Bypas**S S**urgery (PROMISS) derives from current discordant data in the literature regarding the potential inferior graft patency when CABG is performed off-pump as compared with the arrested heart on-pump procedure [12-15]. There would be no advantage to the patient if a short term benefit was to be compromised by the ultimate goal of achieving perfect myocardial revascularisation. In fact, while some studies show no difference in graft patency, others show decreased graft permeability when OPCAB is used [12-15].

Many procedural and surgeon related factors (exposure, stabilisation, shunt use, operator's experience and patient selection) can account for these differences so it is important to evaluate both off and on-pump coronary bypass surgery using standardised techniques, unbiased assessment of results and an accurate patency control method.

Until recently, coronary angiography was the only method to assess coronary artery bypass graft patency. This technique is stressful, causes discomfort and has local (limb ischemia, haematoma, false aneurysm), neurological (stroke, TIA) and cardiac (graft dissection, myocardial infarction, arrhythmias) complications in up to 1% of cases [16]. From an ethical standpoint, a potential life threatening complication risk in asymptomatic patients discouraged us from embarking on a randomized study using conventional coronary angiography.

Recent advances in multidetector computed tomography (MDCT) technology, namely with reduced acquisition time and improved spatial resolution, has permitted its application to the detection of coronary artery disease and bypass graft evaluation [17]. Relative graft immobility and freedom from calcification allows multidetector computed tomography to achieve high diagnostic accuracy for coronary bypass graft patency evaluation [18-20]. This led to the design of a randomized but non invasive study of coronary bypass graft patency, comparing on and off-pump techniques and the conception of PROMISS.

PROMISS primary objective is to compare early coronary bypass graft patency by 16 slice computed tomography in patients with multivessel coronary artery disease undergoing first time coronary artery bypass grafting with (on-pump) and without (off-pump) cardiopulmonary bypass.

Secondary endpoints are:

1- Mortality and morbidity in patients operated with on-pump and off-pump.

2- Comparative changes in neuro-cognitive performance between base line and 4–6 weeks by a battery of neuropsychological tests.

3- Evaluation of functional status, ischaemic threshold by stress test, quality of life and adverse events at 6 months and one year.

Finally, as tertiary end point we aim to analyse hospital costs.

## 2. Methods

PROMISS is a prospective, randomized, single blinded, single centre study.

### 2.1 Organization

Patient inclusion, surgery, post operative care, multidetector computed tomography and follow-up are conducted at Hospital da Cruz Vermelha Portuguesa. The principal investigator designed the study and is the surgeon responsible for performing all on-pump and off-pump operations. The principal investigator has a large experience in on-pump CABG and has performed more than 300 OPCAB surgeries before initiating the study.

### 2.2 Ethical Issues

PROMISS is registered in The International Society for Clinical Randomized Trials (ISRCTN58800729), it is conducted in accordance with the principles of The Declaration of Helsinki, with the Portuguese laws and rules and subscribes to the principles outlined in the International Conference on Harmonisation of Good Clinical Practice [21].

All patients receive full explanation of study objectives, the operations to be performed, with or without CPB according to randomization, its risks and benefits and signed the informed consent form.

Any death during the study period requires the hospital ethical commission to be informed.

### 2.3 Recruiting Process

All patients that comply with the inclusion and exclusion criteria are considered for enrolment in PROMISS (Table 1). Inclusion requires consensus between two surgeons regarding the operative plan: number of grafts with at least three distal anastomoses and target vessels to bypass. This operative plan is then recorded in the patient's case report form before the operation.

**Table 1:** Eligibility Criteria

**Inclusion**	**Exclusion**
Multivessel CAD	IV inotropes, IABP, Assisted Ventilation
First Time CABG with ≥ 3 grafts	Associated Surgical Procedure
Age 30–90 years	Creatinin > 1.5 ULN
Signed Informed Consent	Chronic Atrial Fibrillation
	Iodine Allergy
	Non Menopaused Woman
	Inability to Give Informed Consent

No patient is excluded because of recent myocardial infarction, ventricular dysfunction, or poor quality target vessels (size, location, calcification). No patient is excluded for associated morbid conditions with the exception of renal insufficiency (because of iodine administration) and atrial fibrillation (multidetector computed tomography imperative).

**Figure 1:**
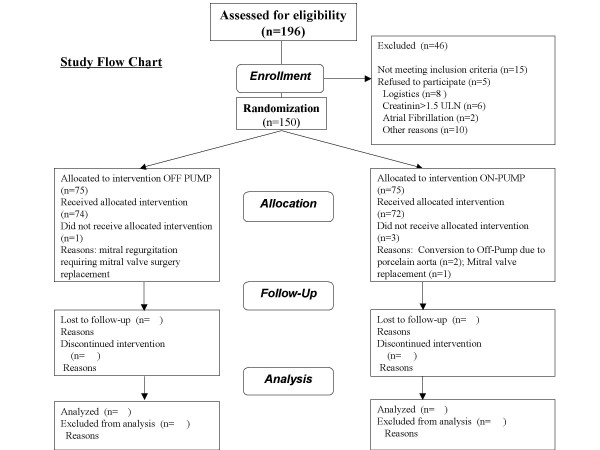
Study Flow Chart.

During the study period, a patient diagram flow is used indicating patients screened, patients enrolled and reasons for exclusion, patients randomized and patients available for MDCT and follow up according to the CONSORT recommendations (Figure 1) [22].

### 2.4 Randomization

The informed consent is signed before randomization. Randomization is performed by the method of random permuted blocks. A sealed envelope containing the patient's number is opened in the operating room before the beginning of the operation.

### 2.5 "Cross-over" and study discontinuation

Cross-over from one group to another is possible in case patient safety is deemed at risk, a condition that is estimated to occur in less than 5% of the cases:

• From on-pump to off-pump group in case of calcified ascending aorta.

• From the off-pump to the on-pump group in case of refractory hemodynamic deterioration.

Patients who cross over from off-pump to on-pump are analysed in the original off-pump group for which he or she was randomized in intent to treat analysis.

Premature exit from the study is possible at any moment at patient's will or because of inclusion/exclusion criteria violation.

### 2.6 Surgical technique and post operative care

Premedication, anaesthetic protocol, patient opening and closing, harvesting and graft anastomoses techniques are the same in both groups.

On-pump CABG uses a conventional roller pump cardiopulmonary bypass machine with an arterial filter, continuous ultra filtration, and a central temperature that is allowed to drift to 34°C. Myocardial protection is provided by 34°C antegrade blood-potassium cardioplegia delivered every 25 minutes.

Off-pump coronary artery bypass grafting uses a deep pericardial stitch and vacuum stabilizers for exposure. Intra coronary shunts are systematically used.

The planned/performed distal anastomoses index and the reason for eventual incomplete revascularisation as well as target coronary artery quality (scale 1–4) are noted in the two groups.

Post operative care is under the responsibility of the same intensive care team and follows the rules and protocols of the hospital ICU.

### 2.7 Blinding

Treatment group is blinded to patients, family and investigators responsible for the MDCT angiographic control, neuro-psychological tests and follow-up.

### 2.8 Data Collection

Demographic data, pre operative, intra operative, post operative and follow up variables are reported in a dedicated Case Report Form. Event adjudication is made by hospital physicians according to standardized definitions (Table 2). Protocol adherence and data collection reliability are monitored by an independent external body verifying conformity with good clinical practice requisites (EuroTrials, Scientific Consultants, Lisbon, Portugal).

**Table 2:** Definition of Post Operative Events

**Outcome**	**Definition**
Mortality	Autopsy result.
Arrythmias	Episodes requiring administration of iv anti arrythmics or electric choc. Atrio-ventricular conduction disturbances defined as requiring temporary or definitive pace maker.
Reoperation	Reoperation or recatheterisation for myocardial ischemia.
Myocardial Infarction	New Q wave or increase in CK-MB > 5xULN.or troponin T > or = 1 ng/ml.
CK-MB and troponin T	Mean values at 6, 12 and 24 hours post operatively.
Cardiac Faillure	Cardiac resuscitation manoeuvres or intra aortic balloon pump. Level of inotropic support.
Neurologic Events	Coma (Glasgow score), stroke (focal neurologic deficit > 24 hours), transient ischemic attack (focal neurologic deficit < 24 hours), delirium.
Respiratory Complications	ARDS, ventilation > 24 hours, pneumonia, thoracic effusion or pneumothorax requiring drainage and PaO2 at discharge from ICU.
Renal Complications	Renal dialysis, ultra filtration, need for continuous iv diuretics, serum creatinin increase between base line and ICU discharge.
Bleeding Complications	Reoperation for bleeding, blood products administration, total blood drainage.
Infectious Complications	Sternal infection (deep and superficial); septicaemia.
Gastro Intestinal Complications	Cholecystitis, Pancreatitis, Mesenteric Ischaemia, GI perforation, GI bleeding, Intestinal Occlusion.
Ressource utilisation	Readmision to ICU; ICU and hospital length of stay.

### 2.9 Assessment of results

#### a/ Principal end point

The study main end point, comparison of graft patency at 4 to 6 weeks between the two groups, is performed by a GE Lightspeed 16 slice multidetector computed tomography using a standard protocol already described [23]. A cardiologist and a radiologist, with previous knowledge of the number and distribution of grafts performed but not the technique that was used assess the grafts using multiplane reformatted images, maximal intensity projection images and three dimensional reconstructions. Quality image is graded as good/excellent, minor artefacts, moderate artefacts and major artefacts. Only good/excellent images and images with minor artefacts are considered for analysis.

Each graft is independently graded using a three grade scale: occluded graft, graft stenosis of 50% or more, and stenosis < 50% or no stenosis at proximal anastomoses (if any), body graft and distal anastomosis [23].

Graft occlusion is defined as absence of contrast material along the course of the graft, through the graft anastomosis to the native coronary artery or to the following graft segment and native vessel. In sequential bypass grafts, each anastomosis of one graft is counted as a separate graft. The overall graft score is the worst of the three subcsores. Graft patency is evaluated according to graft material, site, as well as number of anastomoses patent per patient.

#### b/ Secondary endpoints

Secondary end points are:

1- Revascularisation index: number of planned distal anastomoses/number of performed distal anastomoses.

2- Adverse events at hospital discharge and at 4–6 weeks (Table 2). Each complication is separately analysed, Major adverse events rate is also recorded as the cumulative incidence of mortality, myocardial infarction, stroke or TIA, dialysis, reoperation for ischemia, ventilation > 24 h.

3- Neuro-cognitive assessment

Neurocognitive assessment consisted on the performance at baseline and at 4–6 weeks of the following tests:

• Digit span (from Wechsler Adult Intelligence Scale-III)

• Digit Symbol (from Wechsler Adult Intelligence Scale -III)

• Grooved pegboard test

• Auditory verbal learning test

• Complex Figure Test

• Trail making test A and B

• Letter Word Fluency

• Controlled Oral Word Association (COWA)

• Hospital Anxiety Depression Scale (HADS)

• Beck Depression Inventory

The assessment of 50 healthy demographically matched control subjects, with the same test battery and test-re-test procedures, will enable the calculation of T -scores for each neuropsychological evaluation. Decline will be defined by a drop equal or greater to 15 points.

4- Follow up at 6 months and 1 year will compare functional status, stress test, adverse events and quality of life by EuroQOL between the two groups.

#### c/ Tertiary end point

is the analysis of costs: direct patient care related in hospital costs.

### 2.10. Statistical analysis

#### a/ Sample size

PROMISS was planned as a non inferiority study of one intervention (OPCAB) relative to the reference operation (on-pump). This study was designed with adequate power (80%; α level of 0.05) to detect an absolute difference in patency rates between groups in either direction as small as 5%. The calculation resulted in a total of 426 units (distal graft anastomoses). Since each patient has by the inclusion criteria at least 3 grafts, 71 patients in each group are necessary (213 grafts) for a total of 142 patients. Considering the possibility of 4 patients' withdrawal in each group, a total sample of 150 patients was established.

#### b/ Statistical analysis

Discrete data are presented as numbers and percentages; continuous data are presented as median, mean (SD) and compared using t test or the Mann-Whitney test. Dichotomous morbidity and mortality outcomes are analyzed using the Fisher exact test. Ordered categorical outcomes are compared between groups using the Mantel Haenszel χ^2^.

Dichotomous patency outcomes or graft stenosis > 50% are analyzed using the Fisher exact test. Mean number of patent anstomosis per patient is compared using the t test or the Mann-Whitney test.

## 3. Discussion

The **P**rospective **R**andomized Comparison of **O**ff-Pump and On-Pump Mult**I**-vessel Coronary Artery Bypas**S S**urgery (PROMISS) was designed to try to answer the question of whether or not coronary artery bypass grafting performed off-pump has the same early graft patency as if performed on-pump in arrested hearts.

The sample size was calculated in order to detect a +/- 5% difference between the two techniques. This trial includes only patients with multi vessel disease with an indication to perform at least 3 distal coronary artery anastomoses for two reasons. Firstly to compare the two techniques in terms of completeness of revascularisation since various series have reported a lower number of grafts/patient in OPCAB relative to conventional on-pump surgery [8]. Secondly to compare OPCAB graft patency with the reference method in a high percentage of cases requiring revascularisation of the lateral wall for its reported increased technical difficulty [24,25]. Complete revascularisation is mandatory including the lateral wall since it is one reason for the superior outcomes of CABG compared with percutaneous coronary intervention [26,27].

Few studies have compared OPCAB versus CABG with CPB among randomly assigned patients unselected for coronary anatomy, ventricular function or co-morbidities except renal insufficiency. All surgeries using standardized techniques were performed by a single surgeon experienced with both methods in an attempt to eliminate technical variability.

This is the first randomized study comparing early graft patency in off-pump and on-pump coronary bypass surgery using 16 slice multidetector computed tomography in patients with multivessel disease. Although 64 slice multidetector computed tomography has improved temporal and spatial resolution, there is ample evidence that, with appropriate heart rate reduction, 16 slice MDCT is able to accurately detect graft occlusion and stenosis compared to conventional coronary angiography [23,28,29].

Limitations of this study include the fact that physicians involved in patient care, except those assessing graft patency, are not blinded to treatment method, which can eventually bias treatment decision making and length of stay. Another limitation is the single surgeon type of study which may prevent generalizability of the results. Finally, the assumption made for sample size calculation that graft patency within a patient is an independent research unit may not always apply requiring application of statistical adjustment.

## Conclusion

The Prospective Randomized Study of graft patency in Off-pump and On-pump MultI-Vessel coronary artery bypasS Surgery (PROMISS) using multidetector computed tomography, has the potential to shed new light on the ongoing debate regarding these two techniques and therefore may have implications on the revascularisation strategy.

## List of Abbreviations

ARDS: Acute Respiratory Distress Syndrome; CABG: coronary artery bypass grafting; CAD: Coronary Artery Disease; ICU: Intensive Care Unit; MDCT: Multidetector Computed Tomography; OPCAB: Off-Pump Coronary Artery Bypass; PROMISS: **P**rospective **R**andomized Study of graft patency in **O**ff-pump and On-pump **M**ult**I**-Vessel coronary artery bypas**S S**urgery; TIA: Transient Ischemic Attack; ULN: Upper Limit of Normal of Laboratory value.

## Competing interests

The authors declare that they have no competing interests.

## Authors' contributions

All authors read and approved the final manuscript. Specifically, MSU principal investigator, conceived and designed the study and drafted the manuscript. FM is responsible for performing computed tomography studies and helped draft the manuscript. SC conceived and coordinated the neurocognitive evaluation and helped with statistical analysis. MPM helped draft the manuscript and supervised the research project.
